# Differences between intentional and accidental ingestion of foreign body in China

**DOI:** 10.1186/s12876-020-01224-z

**Published:** 2020-04-06

**Authors:** Ye Zong, Haiying Zhao, Can Sun, Ming Ji, Yongdong Wu, Shutian Zhang, Yongjun Wang

**Affiliations:** Department of Gastroenterology, Beijing Friendship Hospital, Capital Medical University, Beijing, 100050 China

**Keywords:** Foreign body, Intentional ingestion, Upper gastrointestinal tract, Endoscopy

## Abstract

**Backgrounds:**

Previous reports of foreign-body ingestion focused primarily on accidental ingestion and very few studies focused on intentional ingestion of foreign body (FB) in China. Our study aimed to compare the prevalence of different age, gender, types, locations and management of FB ingested between intentional ingestion and accidental ingestion of FB in Northern China.

**Methods:**

A retrospective case series studied all patients with suspected FB ingestion in Digestive Endoscopy Center of Beijing Friendship Hospital, between January 2011 and January 2019. The patients were divided into 2 groups. Group A included the patients who intentionally ingested FBs, and Group B included the patients who accidentally ingested FBs. Patients’ database (demographics, past medical history, characteristics of FB, endoscopic findings and treatments) were reviewed. Statistical analyses were conducted using SPSS software.

**Results:**

Group A consisted of 77 prisoners, 2 suspects and 11 psychologically disabled persons. Group B consisted of 1020 patients with no prisoners, suspects or psychologically disabled persons. In Group A, there were no food-related foreign bodies, and the majority of FBs were metallic objects (54.44%). However in Group B, food-related FBs were the most common (91.37%). In Group A, 58 cases (64.44%) were located in the stomach, while in Group B, 893 cases (87.55%) were located in the esophagus (*P* < 0.05). 1096 patients successfully underwent endoscopic removal and 14 failed, including 9 cases in Group A and 5 cases in Group B. The duration of FBs impaction was longer in Group A than that in Group B (*P* < 0.05).

**Conclusions:**

In our study, the patients who intentionally ingested FB were mainly prisoners, FBs were mostly sharp metallic objects, the duration of FBs impaction was longer, and the rate of successful endoscopic treatment was lower than that of the general population. Attention should be focused on these patients.

## Background

Foreign body (FB) ingestion, including food bolus impaction, is a common clinical problem in China [1, 2]. Although ingestion of FB by adults is generally accidental, the intentional ingestion of FB occurs in some adults such as prisoners [3], patients with psychiatric disorders or mental retardation.

European guidelines reported that 80–90% of ingested FBs can spontaneously pass through the gastrointestinal tract, while 10–20% of ingested FBs may need to be removed endoscopically [4]. However, a recent study reported FBs were encountered at endoscopy in almost half of the cases [5]. The management of FB depended on a number of factors, such as the type, size, shape of the body, time elapsed since ingestion and symptoms or signs of complications. The patients who intentionally ingested FB form a special group. The study reported that the most commonly intentionally ingested objects included razor blades, batteries, and other sharp metallic items, which were not usually reported in the general population, and there was a greater demand for endoscopic and surgical interventions [6].

To our knowledge, very few studies have focused on the intentional ingestion of FBs in China, therefore we aimed to compare the prevalence of different age groups, gender, types of objects, locations and managements of FB ingested between intentional and accidental ingestion groups among Chinese people.

## Methods

### Patients

This retrospective study was approved by the Ethic Committee of Beijing Friendship Hospital. Consecutive adult patients with suspected FBs referred to the Digestive Endoscopy Center of Beijing Friendship Hospital were included in the study from January 2011 to January 2019. The patients whose FBs had passed through the ligament of Treitz or the patients with perforation before endoscopic treatment were not referred to the Digestive Endoscopy Center. The patients were divided into 2 groups, Group A consisted of patients with intentional ingestion of FBs, and Group B consisted of patients with accidental ingestion of FBs.

### Endoscopic procedures

After fasting for 4 to 6 h, each patient underwent an upper endoscopy under local pharyngeal anesthesia with Lidocaine mucilage. Flexible endoscopes (GIF-Q240, GIF-Q260, GIF-H260, GIF-H290; Olympus Optical Co, Ltd., Tokyo, Japan) were used for the procedure. A variety of accessory devices were used to remove the FBs, which included foreign-body retrieval forceps, retrieval baskets and snares. A latex protector hood was used to protect the digestive tract while removing FBs.

### Data collection

Patient demographic data consisting of age, gender, and intention to ingest were analyzed. Clinical features of FBs, consisting of type, sharpness of objects and location, and Endoscopic data, consisting of methods and effectiveness, were all analyzed.

### Statistical analysis

Descriptive statistics included median and interquartile ranges for quantitative variables and proportions for categorical variables. Proportions were compared with chi-square test. Continuous variables were compared with Mann–Whitney U test. *P*-values of less than 0.05 were considered to indicate statistical significance. All of the statistical analyses were conducted using SPSS software, version 22.0 (IBM, Armonk, NY, USA).

## Results

### Differences of gender and age distribution between two groups

From January 2011 to January 2019, a total of 1326 patients with suspected upper gastrointestinal FBs underwent gastroscopy. FBs were confirmed in 1110 cases while no FBs were found in 216 cases. The patients were divided into two groups: there were 90 patients who intentionally ingested foreign body in Group A; there were 1020 patients who accidentally ingested a foreign body in Group B. Group A consisted of 77 prisoners, 2 suspects and 11 psychologically disabled persons, and the ratio of males to females was 79 males (87.78%) to 11 females (12.22%); in Group B, the ratio was 497 males (48.73%) to 523 females (51.27%) with no prisoners, suspects or psychologically disabled persons. There were significant differences in gender distribution between two groups (*P* < 0.05) and the ratio of males to females in Group A was much higher than that in Group B. The differences in the age distribution were observed between the two groups (*P* < 0.05). In Group A, 56 cases (62.2%) were aged between 14 and 44 years, 30 cases (33.33%) between 45 and 59 years, and only 4 cases (4.44%) were over 60 years; in Group B, there were 229 cases (22.45%) aged between 14 and 44 years, 280 cases from 45 to 59 years, and 511 cases (50.10%) over 60 years (Table 1).

**Table 1:** Differences in gender and age distribution between two groups

	Group A^a^		Group B^b^		*P*
Gender	n	%	n	%	
Male	79	87.78	497	48.73	< 0.05
Female	11	12.22	523	51.27	
Age					
14–44	56	62.22	229	22.45	< 0.05
45–59	30	33.33	280	27.45	
≥ 60	4	4.44	511	50.01	

^a^ Group A:the group consisted of patients with intentional ingestion of FBs

^b^Group B: the group consisted of patients with accidental ingestion of FBs

### Differences in the types and location of FBs between the two groups

**Fig. 1:**
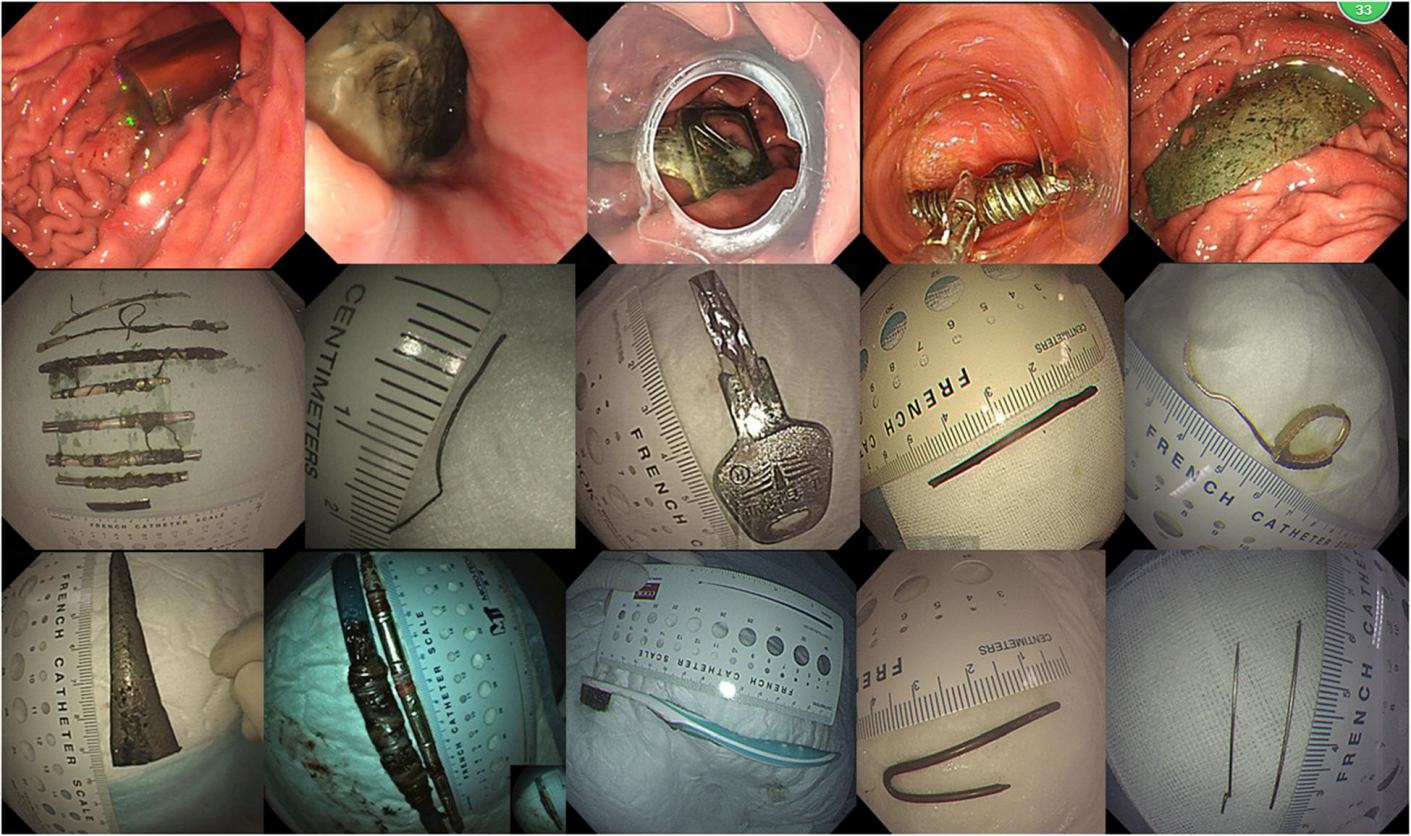
In Group A which consisted of patients with intentional ingestion of FB, FBs included hair, steel wires, other metallic objects, pen or toothbrush and so on, and the majority of FBs were metallic objects

**Fig. 2:**
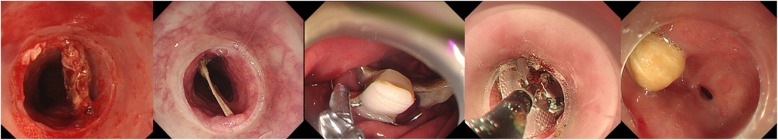
In Group B which consisted of patients with accidental ingestion of FB, FBs included date pits, bones, food bolus, packed tablets, dental prosthesis

The types of FBs in Group A were very different from those in Group B (Table 2). In Group A, there were no food-related FBs, and the majority of FBs were metallic objects (54.44%) (Fig. 1). However in Group B, the majority of FBs were food-related objects (91.37%) (Fig. 2). In Group B, 91 patients (8.92%) with FBs had esophageal diseases, including esophageal cancer (13 cases), postoperative esophageal stenosis (36 cases), cicatricial stenosis caused by corrosives (18 cases), other unexplained stenosis (18 cases), and esophageal diverticulum (6 cases). There were no patients who had esophageal diseases in Group A. The location of FBs was different between the two groups (*P* < 0.05). In Group A, 58 cases (64.44%) were located in the stomach, 16 cases (17.78%) in the esophagus and 16 cases (17.78%) in the duodenum. In Group B, 893 cases (87.55%) were located in the esophagus, 116 cases (11.37%) in the stomach, and 11 cases (1.08%) in the duodenum (Table 3).

**Table 2:** Differences in the types of ingested foreign bodies between the two groups

	Group A^a^		Group B^b^	
Type	n	%	n	%
hair	5	5.56		
Steel wires	19	21.11		
Other metallic objects	52	57.78		
Glass or China pieces	6	6.67		
Pen or toothbrush	5	5.56		
Drug	2	2.22		
Phone shell	1	1.11		
Date pits			430	42.16
Bone			242	23.73
Fish bone			169	16.57
Food bolus			91	8.92
Packed tablets			54	5.29
Dental prosthesis			34	3.33

^a^Group A:the group consisted of patients with intentional ingestion of FBs

^b^Group B: the group consisted of patients with accidental ingestion of FBs

**Table 3:** Differences in the locations of foreign bodies between two groups

	Group A^a^		Group B^b^		*P*
Location	n	%	n	%	
Esophagus	16	17.78	893	87.55	< 0.05
Stomach	58	64.44	116	11.37	
Duodenum	16	17.78	11	1.08	

^a^Group A:the group consisted of patients with intentional ingestion of FBs

^b^Group B: the group consisted of patients with accidental ingestion of FBs

### Endoscopic managements

1096 patients successfully underwent endoscopic removal but this failed in 14 cases, with 9 of these being in Group A and 5 in Group B. Among the 14 cases, 3 were located in the esophagus, 6 in the stomach and 5 in the duodenum (Table 4, Table 5). As for Methods of endoscopic extraction: For Group A, in 47 cases (52.22%) the FBs were removed with foreign body forceps and a foreign body cap, in 28 cases (31.11%) using a snare, and 15 cases (16.67%) using a net basket. For Group B, in 766 cases (75.10%) the FBs were removed with foreign body forceps and a foreign body cap, in 202 cases (19.80%) with snare apparatus, and in 52 cases (5.10%) with a net basket (*P* < 0.05).

**Table 4:** Locations of the foreign body in cases where endoscopic removal failed

	Group A^a^		Group B^b^	
	n	%	n	%
Location				
Esophagus	1	11.11	2	40.00
Stomach	5	55.56	1	20.00
Duodenum	3	33.33	2	40.00
Total	9	100.00	5	100.00

^a^Group A:the group consisted of patients with intentional ingestion of FBs

^b^Group B: the group consisted of patients with accidental ingestion of FBs

**Table 5:** Foreign objects which failed to be removed by endoscopy

Group	Foreign object	
Group A^a^		n
	hair	2
	Steel wires(≥6 cm)	4
	Other metallic objects^c^	3
Group B^b^	Dental prosthesis	3
	Bone	2

^a^Group A:the group consisted of patients with intentional ingestion of FBs

^b^Group B: the group consisted of patients with accidental ingestion of FBs

^c^other metallic objects included steel nail,handle and metal plate

### Differences in the time between FBs ingestion and effective treatments

In Group A, 5 patients who ingested hair retained FBs for the longest time, with an average of 7 to 14 months. Other than these 5 cases, the median time for the remaining patients was 16 h (range 6–89 h), while the median foreign body retention time for Group B was 6 h (range 2–76 h) (*P* < 0.05).

## Discussion

Foreign-body ingestion is a common clinical problem in China. However, there are no reports which focus on Chinese patients who intentionally ingest FBs. In adults, intentional ingestion of FBs commonly occurs among those with psychiatric disorders, pica and prisoners [7, 8]. In our report, the number of patients who intentionally ingested FBs was not large, but the characters of these patients were very different from those who accidentally ingested FBs.

Most of the patients in our study who intentionally ingested FBs were male; however, there was no significant difference between male and female in the general population [2, 9]. Most of the patients with intentional ingestion were prisoners in custody, and they could be considered as seeking secondary gains through access to a medical facility or even manipulative attempts to escape incarceration, which was similar to findings in a previous study [3].

In our report, most of the patients accidentally ingesting FBs were elderly (≥60 years old), which was different from those described in previous reports [2, 10], because the most common foreign body in our study was date pits. The types of FBs ingested were influenced by differences in the characteristics of the study groups, such as age and dietary habits [11]. In Northern China, people’s eating habits include eating pasta; meanwhile, date cakes or soups containing many dates are also very popular. Date pits in Northern China are usually larger than those in Southen China. However the elders’ masticatory function is usually decreased. Therefore, the elders were more likely to ingest this kind of date pit accidentally. Most of the people who intentionally ingested FBs were younger than those who accidentally ingested them in our study.

As for the type of FBs, most of the FBs in the general population were food-related, which was consistent with previous reports [12–14], but in different countries and regions, there were various differences in food related FBs due to different eating habits and types of food. In the group with intentional FBs ingestion, metallic objects are more common, among which steel wire accounts for 21.1% and other metallic materials for 57.8%.In a study of intentional swallowing of FBs conducted at Rhode Island Hospital, Huang and associates [8] found the most commonly ingested FBs were pens (23.6%), batteries (28; 9.2%), knives (22; 7.2%), razor blades (21; 6.9%), other metal objects (20; 6.6%), pencils (19; 6.2%), toothbrushes (18; 5.9%), spoons (15; 4.9%), and coins (13; 4.2%),which also indicated there were no food-related FBs and metal objects were very common among these patients. So X-ray could play an important diagnostic role in this situation [15].

In the general population, most of the FBs were in the esophagus, which was consistent with previous reports [1, 2, 10]. In patients who intentionally ingested FBs, the FBs were usually in the stomach, and the proportion in the duodenum was also higher than that of the general population. A study by Poorvi et al. reviewed 141 episodes of intentional FB ingestion and found most items were also located in the stomach [16]. Swallowing intentionally might help the FBs to pass through the esophagus. However the FBs in patients with intentional ingestion were longer and sharper, so it was more difficult for those to pass through the pylorus. The patients who had intentional ingestion had longer time to present, which allowed an object to transit from the esophagus into the stomach for more time. The patients with accidental ingestion were more likely to have esophageal disease than those with intentional ingestion, which prevented the food from esophagus to the stomach.

Endoscopic intervention remains the preferred modality of foreign-body extraction with a high success rate [16–19]. However the failure rate among patients who intentionally ingest FBs is significantly increased. In our reports, there was a 10% failure rate of endoscopic treatment in the patients who intentionally ingested FBs, while there was only a 0.5% failure rate among patients who accidentally ingested FBs. In our reports, both failure rates were lower than those in previous studies, because the data originated from the Digestive Endoscopy Center. In our hospital, before the patients came to the Digestive Endoscopy Center, they had been previously assessed by the doctor of the emergency, and radiography had been done. The patients who were not suitable for endoscopic treatment were not referred to the Digestive Endoscopy Center. And the patients whose FBs had passed through the ligament of Treitz or the patients with perforation before endoscopic treatment were not refferred to the Digestive Endosccopy Center. Our reports also showed that the failure of endoscopic treatment was related to the type and size of FBs. Long FBs and complicated or sharp FBs were difficult to be removed by endoscopy. In our study, most FBs were able to be removed with FB forceps and a FB cap. We also found that the FB cap was very useful, as it could keep the visual field clear and safely improve the management of sharp FBs [20]. Different endoscopic methods and equipment were used according to the shape, size or sharpness of the FBs. Snares and net baskets were used more frequently in the patients with accidental ingestion. Palta et al [17] reported that the results of their stuy did not allow them to determine which endoscopic accessory worked best for foreign-body removal, and all devices had a high success rate. The choice of endoscopic accessory should be individualized for the specific type of FBs and several different accessories should be available when performing endoscopy in patients with FBs ingestion.

The duration of FBs in upper gastrointestinal tract was different between the patients with intentional ingestion and those with accidental ingestion. Previous research showed that as the time after ingestion increased, the rate of successful FB removal by endoscopy decreased. Long delays between ingestion to presentation and intervention might account for the relatively high rates of surgery, perforation, and mortality [17, 21]. Hong et al. showed that impaction duration and sharpness of FBs were the two important risk factors for the development of major complications and in particular, impaction duration of over 12 h had a 2.4-fold increased risk of major complications [13]. In our study, most of the patients who intentionally ingested FBs were prisoners and they usually took longer time to be noticed than those with accidentally ingested FBs. Sharp metal objects were commonly intentionally ingested by prisoners, which also decreased the rate of successful FB removal by endoscopy. On the contrary, patients who accidentally ingested FBs usually sought for medical help as soon as possible.

## Conclusion

In our study, patients who intentionally ingested FBs were mainly prisoners, FBs were mainly sharp metal objects, the duration of FB impaction was longer, and the rate of successful endoscopic treatment was lower than that of the general population. Attention should be focused on these patients because their intentional deeds are preventable.

## Data Availability

The datasets used and/or analyzed during the current study available from the corresponding author on reasonable request.

## Abbreviation

FB: Foreign body
